# Implications of Elevated Fibrosis-4 Index in Patients Receiving Trans-Catheter Aortic Valve Replacement

**DOI:** 10.3390/jcm10245778

**Published:** 2021-12-10

**Authors:** Teruhiko Imamura, Nikhil Narang, Hiroshi Onoda, Shuhei Tanaka, Ryuichi Ushijima, Mitsuo Sobajima, Nobuyuki Fukuda, Hiroshi Ueno, Koichiro Kinugawa

**Affiliations:** 1 The Second Department of Internal Medicine, University of Toyama, Toyama 930-0194, Japan; ohiro0203@gmail.com (H.O.); stanaka@med.u-toyama.ac.jp (S.T.); ryuryu0702@gmail.com (R.U.); soba1126@yahoo.co.jp (M.S.); nfukuda@med.u-toyama.ac.jp (N.F.); hueno@med.u-toyama.ac.jp (H.U.); kinugawa-tky@umin.ac.jp (K.K.); 2 Advocate Christ Medical Center, Chicago, IL 60453, USA; nikhil.narang@gmail.com

**Keywords:** heart failure, hemodynamics, congestion, aortic valve disease

## Abstract

Background: The prognostic implication of the fibrosis-4 index, which represents the degree of hepatic injury, on patients receiving trans-catheter aortic valve replacement (TAVR) remains unknown. Methods: Patients who underwent TAVR to treat severe aortic stenosis at our institute between 2015 and 2020 were included in this retrospective study and followed for 2 years from the index discharge. The impact of the fibrosis-4 index, which was calculated using age, hepatic enzymes, and platelet count, on 2-year heart failure readmissions was investigated. Results: A total of 272 patients (median age 85 (82, 88) years old, 76 (28%) men) were included. The median baseline fibrosis-4 index was 2.8 (2.2, 3.7). A high fibrosis-4 index (>3.79) was associated with higher cumulative incidence of the primary endpoint (18% versus 4%, *p* < 0.001) and higher event rates (0.1041 versus 0.0222 events/year, *p* < 0.001), with an adjusted hazard ratio of 1.27 (95% confidence interval 1.04–1.54, *p* = 0.019). Conclusion: an elevated fibrosis-4 index at baseline, indicating the existence of persistent hepatic congestion, was associated with incidences of heart failure following TAVR. Calculating the fibrosis-4 index before TAVR is highly encouraged for risk stratification and shared decision making.

## 1. Background

Survival following trans-catheter aortic valve replacement (TAVR) has improved considerably partly due to improvements in peri-procedural management and patient selection optimization [1,2]. Nevertheless, adverse events after TAVR can occur, including decompensated heart failure [3,4]. It is clinically challenging to ascertain predictive factors that are associated with heart failure recurrence following TAVR given the complex interplay of valvular and intrinsic myocardial dysfunction.

The fibrosis-4 index is a calculated metric used originally to assess the risk of hepatic fibrosis progression in patients with hepatitis [5]. This index is calculated using four standard clinical parameters associated with hepatic function: {[glutamic oxaloacetic transaminase (IU/L)] × [age (years)]}/{[platelet counts (×10^9^/L) × [glutamic pyruvic transaminase (IU/L)]^1/2^} [5]. Recently, the fibrosis-4 index has been used in patients with chronic heart failure [6], where an elevated fibrosis-4 index may be associated with systemic congestion and worse clinical prognosis [7].

This study aimed to investigate the association between the fibrosis-4 index and risk of heart failure recurrence following TAVR.

## 2. Methods

### 2.1. Patient Selection

Patients who underwent TAVR at our institute between 2015 and 2020 were included in this retrospective study. All patients were followed from index discharge for 2 years or until Jun 2021. Written informed consent was obtained from all participants on admission. The institutional review board approved the study protocol.

### 2.2. TAVR Procedure

Patients with severe aortic stenosis with max velocity > 4.0 m/s, mean pressure gradient > 40 mmHg, or aortic valve area < 1.0 cm^2^ were considered for TAVR in a multidisciplinary heart-valve team conference.

All patients received TAVR according to the standard procedure. Patients received self-expandable valves (Corevalve, Evolut R, Evlolut PRO, or Evolut PRO+; Medtronic plc., Minneapolis, Minnesota) or balloon-expandable valves (Sapien XT or Sapien 3; Edwards Lifesciences Inc., Irvine, CA, USA) via trans-femoral, trans-aorta, trans-subclavian, or direct aorta approach under general or local anesthesia support.

### 2.3. Clinical Variables

Demographic, laboratory, echocardiographic, hemodynamics, and medication data within one week before TAVR were collected. Laboratory, echocardiographic, and medication data following TAVR were also collected.

The baseline fibrosis-4 index was calculated as an independent variable according to the following formula: {[glutamic oxaloacetic transaminase (IU/L)] × [age (years)]}/{[platelet counts (×10^9^/L) × [glutamic pyruvic transaminase (IU/L)]^1/2^} [5]. The fibrosis-4 index was calculated also following TAVR.

### 2.4. Clinical Outcomes

All patients were followed up at our institute or affiliated institutes. Heart failure readmissions that required IV diuretics or any other intensive therapies under in-hospital observation were defined as the primary endpoint. All-cause death was also counted. The plasma B-type natriuretic peptide level at one-year follow-up was collected.

### 2.5. Statistical Analysis

Continuous variables were presented as a median and an interquartile range and compared using the Mann–Whitney U test. Categorical variables were presented as numbers and percentages and compared using Fisher’s exact test. A two-tailed *p* < 0.05 was considered statistically significant. Statistical analyses were performed using SPSS Statistics 22 (SPSS Inc, Armonk, IL, USA) and ESR (Ver 1.54; Saitama Medical Center Jichi Medical University, Saitama, JPN).

The primary endpoint was two-year heart failure readmissions. A time-dependent receiver operating characteristics analysis was conducted to calculate the cutoff for the baseline fibrosis-4 index at the primary endpoint using a Youden index. The patient cohort was stratified into two groups using this cutoff.

Cumulative incidence comparisons with a Gray test between the two groups were performed by accounting for death as a competing risk. Negative binomial regression analyses were performed to compare event rates between the two groups. Fine-Gray proportional hazard ratio regression analyses were performed to investigate the impact of the baseline fibrosis-4 index on the primary endpoint by adjusting for potential baseline characteristics that were significantly different in the comparison study (age, body surface area, hemoglobin, plasma B-type natriuretic peptide, and peak velocity at aortic valve).

## 3. Results

### 3.1. Baseline Characteristics

**Figure 1:**
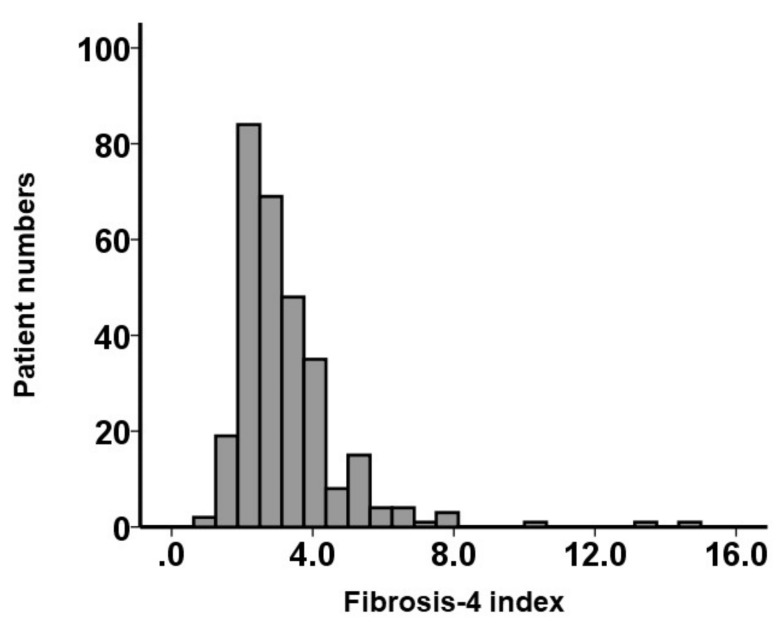
Distribution of the baseline fibrosis-4 index.

A total of 272 patients were included (Table 1). Median age was 85 (82, 88) years old, and 28% were men. The median plasma B-type natriuretic peptide level was 271 (123, 556) pg/mL. The median left ventricular ejection fraction was 64% (54%, 70%). The median peak velocity at aortic valve was 4.5 (4.0, 4.9) m/s. The median STS score was 5.3 (4.2, 7.7), and the median EURO II score was 3.5 (2.4, 4.6). The median fibrosis-4 index was 2.8 (2.2, 3.7). The distribution of the fibrosis-4 index at baseline is displayed in Figure 1.

### 3.2. Post-Procedural Data

Following TAVR, the median plasma B-type natriuretic peptide was 127 (86, 271) pg/mL. The median peak velocity at aortic valve was 2.1 (1.8, 2.4) m/s. All other clinical parameters are displayed in Table 2.

### 3.3. Baseline Fibrosis-4 Index and Heart Failure Readmissions

**Figure 2:**
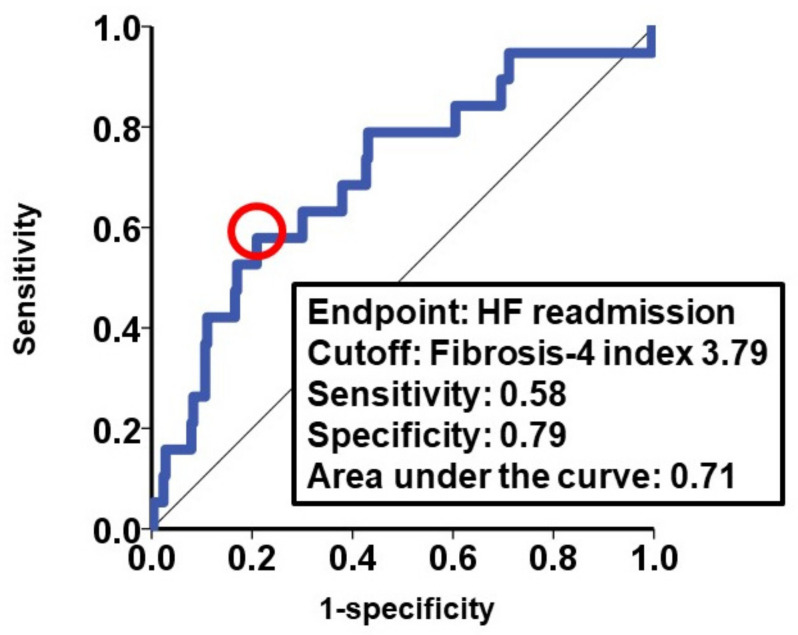
Cutoff of fibrosis-4 index for the primary endpoint. HF, heart failure. Cutoff was calculated as 3.79 of the fibrosis-4 index.

The cutoff of the baseline fibrosis-4 index associated with the primary endpoint was calculated as 3.79 (Figure 2). Patients with a high fibrosis-4 index (>3.79) were older, had lower hemoglobin levels, and had higher plasma B-type natriuretic peptide levels than the low fibrosis-4 index group (*p* < 0.05 for all; Table 1). Following TAVR, those with a high fibrosis-4 index had a lower estimated glomerular filtration ratio and higher plasma B-type natriuretic peptide (*p* < 0.05 for both; Table 2).

**Figure 3:**
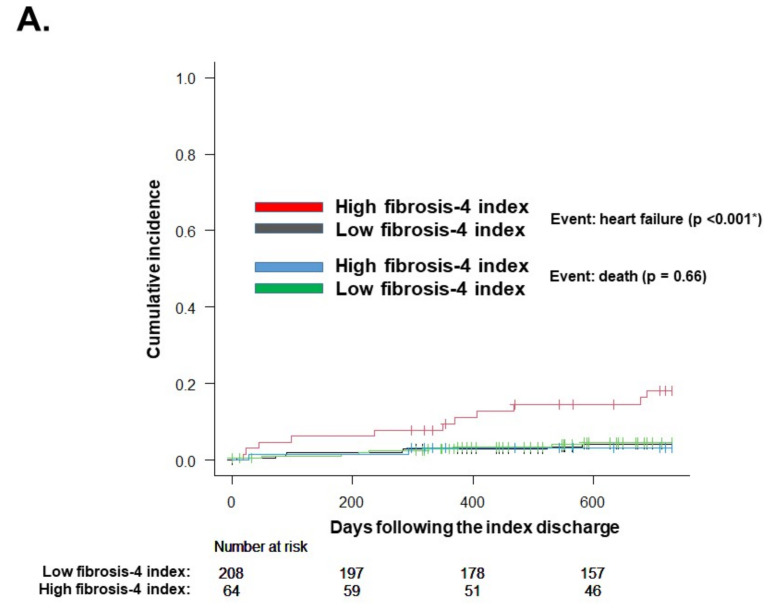

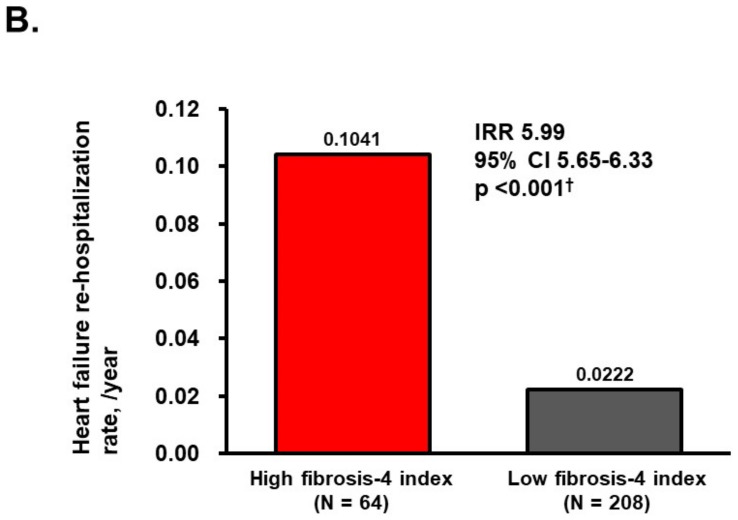
Cumulative incidence of the primary endpoint accounting for death as a competing risk (**A**) and event rates of the primary endpoint (**B**) stratified by the fibrosis-4 index levels. IRR, incidence rate ratio; CI, confidence interval. * *p* < 0.05 by log-rank test. † *p* < 0.05 by negative binomial regression analysis.

During the observational period following the index discharge for a median of 730 (704, 730) days, there were 11 and 8 heart failure readmissions in the high and low fibrosis-4 index groups, respectively. Most of them (15/19) were preserved ejection fraction, and others were mildly reduced ejection fraction (3/19) or reduced ejection fraction (1/19). A 2-year cumulative incidence of the primary endpoint was higher in the high fibrosis-4 index group (18% versus 4%, *p* < 0.001; Figure 3A). The event rates of the primary endpoint were higher in the high fibrosis-4 index group (0.1041 versus 0.0222 events/year), with an incidence rate ratio of 5.99 (95% confidence interval 5.65–6.33, *p* < 0.001; Figure 3B).

The impact of baseline fibrosis-4 index on the incidence of primary endpoint was calculated as a hazard ratio 1.27 (95% confidence interval 1.04–1.54, *p* = 0.019), which was adjusted for five potential confounders that were significantly different in the comparison analysis (age, body surface area, hemoglobin, plasma B-type natriuretic peptide, and peak velocity at aortic valve) (Table 3).

### 3.4. Other Clinical Outcomes

At one-year follow-up, median plasma B-type natriuretic peptide did not significantly differ between the two groups (100 (71, 165) pg/mL versus 87 (42, 164) pg/mL, *p* = 0.15). There were 4 and 16 deaths in the high and low fibrosis-4 index groups, respectively. The 2-year cumulative mortality rate did not significantly differ between the two groups (4% versus 5%, *p* = 0.66) (Figure 3A).

### 3.5. Changes in Fibrosis-4 Index Following TAVR

**Figure 4:**
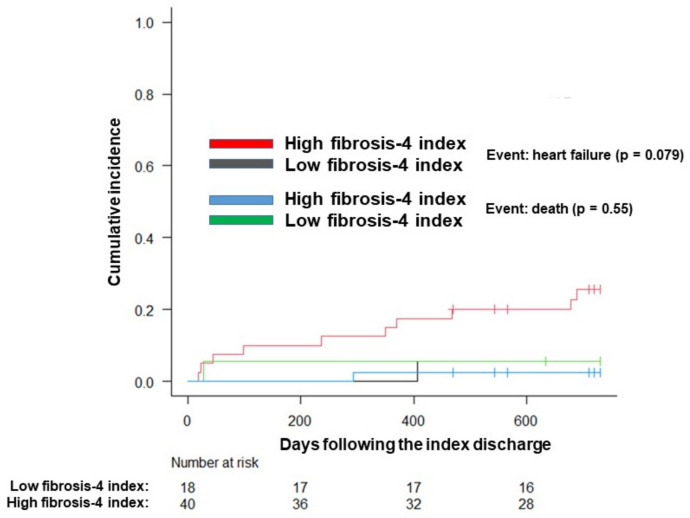
Cumulative incidence of the primary endpoint accounting for death as a competing risk among those with a high fibrosis-4 index at baseline.

Among the 64 patients with high baseline fibrosis-4 indexes, 58 had fibrosis-4 indexes that were also obtained at index discharge. Of them, 18 patients (31%) achieved decreases in their fibrosis-4 index below the cutoff level (<3.79) at index discharge. There were no significant differences in the baseline characteristics between the two groups (decreased and persistently high groups), except for a higher max velocity at the aortic valve in the group with decreases in their fibrosis-4 index (*p* = 0.010; Table 4). The group with a decreased fibrosis-4 indexes tended to be associated with a lower cumulative incidence of the primary endpoint (6% versus 26%, *p* = 0.079; Figure 4). Mortality was not significantly different between the two groups (5% versus 3%, *p* = 0.55).

## 4. Discussion

In this study, we investigated the impact of the baseline fibrosis-4 index on heart failure readmissions following TAVR. We observed that (1) the baseline fibrosis-4 index was elevated in patients with severe aortic stenosis and was associated with more advanced heart failure; (2) the baseline fibrosis-4 index was associated with an increased incidence of heart failure readmissions following TAVR, even after adjustment for other potential confounders; and 3) the peri-procedural improvement in fibrosis-4 index tended to be associated with a lower incidence of heart failure readmissions.

### 4.1. Fibrosis-4 Index and Aortic Stenosis

The fibrosis-4 index was originally introduced to predict the progression of hepatic fibrosis in patients with hepatic diseases [5]. The use of this index has recently expanded to assess the degree of right-sided volume overload and to predict prognosis in patients with acute/chronic heart failure [6,7,8], pulmonary hypertension due to left heart disease [9], and atrial fibrillation [10]. The fibrosis-4 index would reflect hepatic injury via systemic congestion, mostly due to hepatic congestion, and hypo-perfusion due to low cardiac output.

This is the first study to investigate the fibrosis-4 index in patients with aortic stenosis. The median value was 2.8, which is almost comparable with other previous studies including heart failure cohorts [6,7]. Of note, in a study by Takae and colleagues, a higher fibrosis-4 index was associated with clinical outcomes in patients with diastolic heart failure but not in patients with systolic heart failure [7]. They hypothesized that the underlying mechanisms that explained their findings would be systemic tissue fibrosis with a similar pathophysiology between the heart and the liver. A similar pathophysiology might exist in our cohort with aortic stenosis, which often accompanies diastolic heart failure with myocardial fibrosis.

In our study, those with a higher fibrosis-4 index had incremental plasma levels of B-type natriuretic peptide and dilutional anemia, both of which may suggest greater systemic volume overload and more advanced heart failure. Greater impairment of renal function might reflect glomerular congestion due to cardio-renal syndrome in patients with a higher fibrosis-4 index after TAVR. Given these findings, we strongly encourage clinicians to calculate the fibrosis-4 index in all patients with aortic stenosis to assess the refractory systemic congestion and the degree of heart failure progression.

### 4.2. Prognostic Implication of Fibrosis-4 Index

Recurrent heart failure following TAVR has negative prognostic implications [4]. Elevations in intra-cardiac filling pressures and the existence of refractory pulmonary hypertension are associated with poor outcomes following TAVR [11]. The existence of advanced tricuspid regurgitation is also independently associated with poor outcomes after TAVR [12]. Collectively, it is not surprising that a baseline elevated fibrosis-4 index, probably indicating these clinical conditions, was associated with recurrent heart failure following TAVR. Beyond the assessment of heart failure severity, we highly encourage clinicians to calculate the fibrosis-4 index for risk stratification and appropriate shared decision making for all TAVR candidates, particularly the elderly cohort. Some patients who are older with advanced frail and multiple comorbidities might not select TAVR following repeat shared decision making if their calculated fibrosis-4 index is high.

A post-procedural improvement in fibrosis-4 index tended to be associated with a lower incidence of heart failure recurrence. We also showed this important finding in the shared decision-making process. Although further studies are needed to establish how to improve the fibrosis-4 index, aggressive therapeutic approaches using neuro-hormonal blockers and diuretics might be promising tools.

### 4.3. Limitations

This is a retrospective study consisting of a moderate sample size. We observed the association between baseline fibrosis-4 index levels and heart failure recurrence. Detailed mechanisms that explain the causality remains uncertain. We cannot draw any conclusions between fibrosis-4 index levels and mortality due to the low total number of deaths following TAVR. We performed multivariate analyses but other unadjusted potential confounders may also have impacted the risk of the primary endpoint. Patients with liver cirrhosis did not receive TAVR and were not included in this study. Sarajlic and colleagues demonstrated that the sex-specific gene expression was associated with the valvular phenotypes [13]. The uneven sex proportion in our study might have also biased our findings.

## 5. Conclusions

The baseline elevated fibrosis-4 index, which is often associated with systemic congestion, was associated with increased heart failure incidence following TAVR. We highly encourage clinicians to calculate the fibrosis-4 index for all TAVR candidates for risk stratification and shared decision making. An aggressive intervention to decrease the score, including neuro-hormonal blockers and diuretics, might improve the clinical outcomes following TAVR.

## Figures and Tables

**Table 1:** Pre-procedural baseline characteristics.

	Total (*N* = 272)	High Fibrosis-4 Index (*N* = 64)	Low Fibrosis-4 Index (*N* = 208)	*p*-Value
Demographics				
Age, years	85 (82, 88)	86 (83, 90)	85 (82, 88)	0.042 *
Men	76 (28%)	15 (23%)	61 (29%)	0.23
Body surface area, m^2^	1.39 (1.29, 1.52)	1.36 (1.24, 1.48)	1.40 (1.30, 1.53)	0.016 *
Systolic blood pressure, mmHg	114 (103, 125)	110 (100, 122)	114 (104, 126)	0.057
Heart rate, bpm	71 (63, 79)	67 (61, 78)	72 (64, 80)	0.082
Comorbidity				
Atrial fibrillation	28 (10%)	8 (13%)	20 (10%)	0.22
Coronary artery disease	73 (27%)	18 (28%)	55 (26%)	0.44
Chronic obstructive pulmonary disease	19 (7%)	3 (5%)	16 (8%)	0.32
History of stroke	43 (16%)	12 (19%)	31 (15%)	0.27
Peripheral artery disease	81 (30%)	21 (33%)	60 (29%)	0.31
Diabetes mellitus	51 (19%)	7 (11%)	44 (21%)	0.069
Laboratory data				
Hemoglobin, g/dL	11.1 (10.0, 12.2)	10.5 (9.7, 12.2)	11.3 (10.1, 12.2)	0.038 *
Serum albumin, g/dL	3.8 (3.5, 4.0)	3.7 (3.5, 3.9)	3.8 (3.5, 4.0)	0.16
Serum sodium, mEq/L	140 (139, 142)	140 (139, 141)	140 (139, 142)	0.76
eGFR, mL/min/1.73m^2^	48.0 (36.6, 64.0)	46.5 (38.0, 59.5)	49.6 (36.0, 65.4)	0.17
Plasma B-type natriuretic peptide, pg/mL	271 (123, 556)	334 (171, 648)	233 (111, 489)	0.007 *
Echocardiography				
Left ventricular end-diastolic diameter, mm	45 (41, 51)	45 (42, 53)	45 (41, 50)	0.80
Left ventricular ejection fraction, %	64 (54, 70)	63 (54, 70)	64 (54, 70)	0.98
Moderate or greater MR	22 (8%)	6 (9%)	16 (8%)	0.43
Moderate or greater TR	7 (3%)	0 (0%)	7 (3%)	0.15
Aortic valve parameter				
Peak velocity, m/s	4.5 (4.0, 4.9)	4.6 (4.2, 5.0)	4.5 (4.0, 4.9)	0.008 *
Mean pressure gradient, mmHg	48 (38, 59)	53 (43, 63)	46 (37, 58)	0.007 *
Hemodynamics				
Mean right atrial pressure, mmHg	5 (3, 7)	5 (3, 8)	5 (3, 7)	0.88
Mean pulmonary artery pressure, mmHg	19 (16, 23)	21 (16, 25)	19 (16, 23)	0.14
Pulmonary capillary wedge pressure, mmHg	12 (9, 15)	12 (8, 17)	12 (9, 15)	0.50
Cardiac index, L/min/1.73m^2^	2.7 (2.4, 3.0)	2.6 (2.4, 2.9)	2.8 (2.4, 3.1)	0.11
Score				
STS score	5.3 (4.2, 7.7)	5.7 (4.5, 8.5)	5.2 (4.1, 7.3)	0.13
EURO II score	3.5 (2.4, 4.6)	3.6 (2.7, 4.5)	3.5 (2.4, 4.7)	0.71
Medication				
Beta blocker	86 (32%)	18 (28%)	68 (33%)	0.29
Angiotensin converting enzyme inhibitor	45 (17%)	11 (17%)	34 (16%)	0.49
Mineralocorticoid receptor antagonist	75 (28%)	22 (34%)	53 (25%)	0.11
Loop diuretics	158 (58%)	39 (61%)	119 (57%)	0.37
Fibrosis-4 index	2.8 (2.2, 3.7)	4.6 (4.0, 5.5)	2.5 (2.1, 3.1)	<0.001 *

eGFR, estimated glomerular filtration ratio; MR, mitral regurgitation; TR, tricuspid regurgitation. Continuous variables are presented as median and interquartile and compared using the Mann–Whitney U test. Categorical variables are presented as numbers and percentages and compared using the Fischer’s exact test. * *p* < 0.05.

**Table 2:** Post-procedural clinical data.

	High Fibrosis-4 Index (*N* = 64)	Low Fibrosis-4 Index (*N* = 208)	*p*-Value
Laboratory data			
Hemoglobin, g/dL	10.1 (9.6, 11.0)	10.3 (9.7, 11.0)	0.15
Serum albumin, g/dL	3.4 (3.0, 3.5)	3.4 (3.2, 3.7)	0.30
Serum sodium, mEq/L	140 (137, 141)	139 (138, 141)	0.93
eGFR, mL/min/1.73m^2^	46.4 (36.0, 60.7)	52.7 (38.4, 66.4)	0.046 *
Plasma B-type natriuretic peptide, pg/mL	127 (86, 271)	109 (54, 216)	0.013 *
Echocardiography (*N* = 242)			
Left ventricular end-diastolic diameter, mm	45 (41, 52)	45 (42, 49)	0.90
Left ventricular ejection fraction, %	63 (58, 70)	66 (57, 73)	0.37
Moderate or greater MR	0/54 (0%)	8/188 (4%)	0.13
Moderate or greater TR	2/55 (4%)	5/186 (3%)	0.50
Aortic valve parameter (*N* = 242)			
Peak velocity, m/s	2.1 (1.8, 2.4)	2.1 (1.7, 2.3)	0.53
Mean pressure gradient, mmHg	9 (7, 13)	10 (6, 12)	0.47
Medication			
Beta blocker	18 (28%)	69 (33%)	0.30
Angiotensin converting enzyme inhibitor	11 (17%)	42 (20%)	0.32
Mineralocorticoid receptor antagonist	22 (34%)	52 (25%)	0.09
Loop diuretics	30 (47%)	85 (41%)	0.17

Abbreviations are the same as in Table 1. Continuous variables are presented as median and interquartile and compared using the Mann–Whitney U test. Categorical variables are presented as numbers and percentages and compared using the Fischer’s exact test. * *p* < 0.05.

**Table 3:** Impact of the baseline fibrosis-4 index on the primary endpoint.

	Hazard Ratio (95% Confidential Interval)	*p*-Value
Univariate analysis		
Fibrosis-4 index	1.34 (1.13–1.59)	<0.001 *
Multivariate analysis		
Fibrosis-4 index adjusted for five potential confounders	1.27 (1.04–1.54)	0.019 *

* *p* < 0.05 by Fine-Gray hazard ratio regression analysis. For the adjustment, potential confounders that were significantly different in Table 1 were used: age, body surface area, hemoglobin, plasma B-type natriuretic peptide, and max velocity at aortic valve.

**Table 4:** Baseline characteristics among those with a high baseline fibrosis-4 index.

	Persistently High Fibrosis-4 Index (*N* = 40)	Decrease in Fibrosis-4 Index (*N* = 18)	*p*-Value
Demographics			
Age, years	85 (82, 88)	86 (84, 91)	0.11
Men	11 (28%)	2 (11%)	0.16
Body surface area, m^2^	1.36 (1.23, 1.49)	1.30 (1.24, 1.37)	0.26
Systolic blood pressure, mmHg	111 (93, 121)	109 (99, 115)	0.65
Heart rate, bpm	66 (58, 76)	69 (65, 78)	0.082
Comorbidity			
Atrial fibrillation			
Coronary artery disease	14 (35%)	3 (17%)	0.13
Chronic obstructive pulmonary disease	1 (3%)	2 (11%)	0.23
History of stroke	10 (25%)	1 (6%)	0.071
Peripheral artery disease	15 (38%)	5 (28%)	0.34
Diabetes mellitus	3 (8%)	4 (22%)	0.13
Laboratory data			
Hemoglobin, g/dL	10.4 (9.5, 12.6)	10.2 (9.6, 11.1)	0.37
Serum albumin, g/dL	3.7 (3.4, 4.0)	3.8 (3.5, 3.9)	0.97
Serum sodium, mEq/L	141 (139, 142)	140 (139, 141)	0.97
eGFR, mL/min/1.73m^2^	42.3 (31.9, 51.5)	47.0 (39.2, 61.2)	0.10
Plasma B-type natriuretic peptide, pg/mL	338 (165, 624)	451 (213, 736)	0.31
Echocardiography			
Left ventricular end-diastolic diameter, mm	45 (41, 53)	45 (41, 53)	0.93
Left ventricular ejection fraction, %	66 (54, 73)	64 (61, 68)	0.80
Moderate or greater MR	4 (10%)	2 (11%)	0.60
Moderate or greater TR	0	0	-
Aortic valve parameter			
Peak velocity, m/s	4.5 (4.2, 4.8)	5.0 (4.8, 5.6)	0.010 *
Mean pressure gradient, mmHg	49 (43, 58)	62 (54, 81)	0.011 *
Hemodynamics			
Mean right atrial pressure, mmHg	5 (4, 7)	4 (2, 7)	0.27
Mean pulmonary artery pressure, mmHg	23 (15, 26)	19 (14, 21)	0.27
Pulmonary capillary wedge pressure, mmHg	14 (8, 19)	10 (7, 13)	0.12
Cardiac index, L/min/1.73m^2^	2.64 (2.39, 2.92)	2.56 (2.14, 2.68)	0.25
Score			
STS score	6.2 (4.4, 8.8)	5.3 (4.7, 7.4)	0.90
EURO II score	3.7 (2.2, 4.5)	3.7 (3.3, 4.5)	0.84
Medication			
Beta blocker	10 (25%)	7 (39%)	0.21
Angiotensin converting enzyme inhibitor	7 (18%)	3 (17%)	0.63
Mineralocorticoid receptor antagonist	15 (38%)	7 (39%)	0.55
Loop diuretics	28 (70%)	11 (61%)	0.40

Abbreviations are the same as in Table 1. Continuous variables are presented as median and interquartile and compared using the Mann–Whitney U test. Categorical variables are presented as numbers and percentages and compared using the Fischer’s exact test. * *p* < 0.05. Among the 64 patients with high baseline fibrosis-4 indexes, 6 were excluded due to a lack of data.

## Data Availability

Data are available from the corresponding author upon reasonable requests.
